# Autoimmune polyglandular syndrome among Chinese celiac disease patients: a survey of 243 individuals in China

**DOI:** 10.3389/fimmu.2025.1606237

**Published:** 2025-07-08

**Authors:** Shenglong Xue, Yan Feng, Tian Shi, Halina Halike, Ayinuer Maimaitireyimu, Adilai Abudurexiti, Jinjin Xie, Shanxia Yao, Feng Gao

**Affiliations:** ^1^ College of Life Science and Technology, Xinjiang University, Urumqi, China; ^2^ Department of Gastroenterology, People’s Hospital of Xinjiang Uygur Autonomous Region, Urumqi, China; ^3^ Xinjiang Clinical Research Center for Digestive Diseases, Urumqi, China; ^4^ Xinjiang Medical University, Urumqi, China

**Keywords:** celiac disease, autoimmune polyglandular syndromes, epidemiology, autoimmune thyroid disease, glandular autoimmunity

## Abstract

**Background:**

Celiac disease (CeD), an autoimmune enteropathy, is often associated with multiple glandular autoimmune diseases. However, the prevalence and staging characteristics of autoimmune polyglandular syndrome (APS) among CeD patients remain unclear. The aim of this study was to assess the prevalence and clinical features of APS among Chinese CeD patients.

**Methods:**

Clinical data and medical records of 243 CeD patients diagnosed in northwest China were retrospectively analyzed to identify comorbid autoimmune diseases among CeD patients. Serum interferon-ω1, interferon-α, and thyroid autoantibodies (TPOAb and TgAb) were measured, and AIRE mutations were detected. APS typing was conducted based on serum antibodies, gene sequencing (AIRE mutation analysis), and comorbidity analysis.

**Results:**

The overall prevalence of APS in CeD patients was 10.3% (25/243), and the prevalence of different types of APS varied as follows: APS-1: 0.4%, APS-2: 0.4%, APS-3: 8.2%, and APS-4: 1.2%. The prevalence of APS in CeD patients was significantly higher than that in the general population, especially the prevalence of APS-3. Patients with CeD combined with APS had a higher prevalence of vitamin D deficiency (13 of 25 patients, 52%) and *H. pylori* infection (8 of 25 patients, 32%). In addition, CeD patients with combined APS were more likely to have anxiety and depressive symptoms (*P* < 0.05), but there were no significants differences in gender, ethnicity, or body mass index.

**Conclusion:**

This study is the first to systematically evaluate the prevalence and staging characteristics of APS among CeD patients, thereby filling the epidemiological data gap in this area. We emphasize the significance of screening for APS among CeD patients to enable early detection and treatment of associated autoimmune diseases and enhance patients’ quality of life.

## 1 Introduction

Autoimmune polyglandular syndromes (APS) are rare and orphan diseases that are usually characterized by the presence of two or more endocrine gland disorders in the same patient, and in some cases by the combination of other non-endocrine gland autoimmune disorders (1). APS is characterized by a wide range of heterogeneity, which manifests itself sequentially over the time intervals of disease onset (2). Based on the different combinations of autoimmune endocrine diseases, APS has been classified into four types, APS-1, APS-2, APS-3, and APS-4 (2).

APS-1, also known as Autoimmune Poly-Endocrine-Candidiasis-Ectodermal-Dystrophy (APECED) or multiple autoimmune syndrome type 1 (MAS-1), is mainly caused by mutations in the autoimmune regulatory gene (AIRE) on chromosome 21 and is inherited in an autosomal recessive inheritance pattern (3). APS-1 is clinically characterized by chronic mucocutaneous candidiasis (CMC), chronic hypoparathyroidism, and Addison’s disease (AD), as well as ectodermal dystrophy and many other disorders affecting endocrine and non-endocrine organs. APS-1 is diagnosed when an individual has at least two of the three conditions: CMC, hypoparathyroidism, and AD. The diagnostic criteria for APS-1 also include serum interferon-ω1 (IFNω1), the hallmark antibody for APS-1, and interferon-alpha (IFN-α), in combination with mutations in the AIRE gene (4, 5). The prevalence of APS-1 typically ranges from 1:80,000 to 1:100,000, and it can be higher in some populations. For example, it is 1:9,000 among Iranian Jews, 1:14,500 in Sardinia, and 1:25,000 in the Finnish population (6).

APS-2 is defined as AD combined with type 1 diabetes mellitus (T1D) and/or autoimmune thyroid disease (AITD). AD is a mandatory component of APS-2, while T1D and AITD are not. Additionally, many other autoimmune diseases may also be associated with APS-2, including celiac disease (CeD), vitiligo, alopecia areata, myasthenia gravis, pernicious anemia, IgA deficiency, autoimmune liver disease, and hypogonadotropic hypogonadism (7, 8). It is also strongly associated with CeD. The prevalence of APS-2 is approximately 1:20,000, with a male-to-female ratio of 1:3. It has also been shown that APS-2 is more common in middle-aged women (9).APS-3 is the most common subtype, with an approximate prevalence of 1:20,000 in the general population. It also has a higher prevalence in women than in men (10). APS-3 is defined as an association of AITD with other autoimmune diseases, excluding Addison’s disease and hypoparathyroidism (11). APS-4 is a highly heterogeneous disease, and there are no clear and comprehensive diagnostic criteria for it. A study by Elisa Gatta et al. demonstrated a prevalence rate of 9:100,000 in the general population (12). APS-4 is defined as a condition that excludes the endocrine-organ-affecting diseases from the previous groups (11). It involves various clinical combinations of autoimmune diseases, where one endocrine organ (excluding AD, AITD, or hypoparathyroidism) is affected in combination with at least one other endocrine or non-endocrine organ (12).

CeD is a distinct intestinal autoimmune disorder triggered by the ingestion of gluten-containing foods among carriers of the genetically susceptible genes (HLA-DQ2/8), which may result in inflammation, villous atrophy, crypt hyperplasia, and malabsorption in the small intestine (13). When individuals consume gluten-containing foods, it initiates a T-cell-mediated self-destructive process in the small intestinal mucosa, which typically recovers when these grains and gluten are strictly excluded from the diet (14). Currently, a strict gluten-free diet (GFD) remains the sole effective treatment for CeD (15, 16). The prevalence of CeD in the general population varies from 0.5% to 2%, with an average prevalence of about 1%. The clinical manifestations of CeD are diverse (13, 17). Patients with CeD frequently experience gastrointestinal distress, weight loss, fatigue, bone hypomineralization, and hypocalcemia, while only a small proportion of patients present with anemia, weight loss, and infertility (18–20). Moreover, patients with CeD frequently have comorbidities with various extra-intestinal autoimmune diseases, mainly including AITD, T1D, systemic lupus erythematosus, and autoimmune liver disease (21). Deficiencies in iron, zinc, vitamin D, vitamin B12, or folate are the most common laboratory findings (18). Multiple studies have demonstrated that patients with CeD exhibit a high prevalence of glandular autoimmune diseases (22, 23). Autoimmune diseases frequently comorbid with CeD are also listed in the APS diagnostic guidelines. It has been suggested that there may be a high prevalence of APS in patients with CeD. However, the frequency of APS in patients with CeD remains unknown. Globally, there is a lack of epidemiological data on the comorbidity of APS in CeD, and its clinical features and staging distribution still need to be clarified.

In this study, by screening the clinical data and medical records of patients diagnosed with CeD in northwest China, we determined the prevalence of different types of APS in CeD and the clinical features of CeD combined with APS, aiming to provide a basis for the subsequent diagnosis and prevention of APS. This is the first systematic evaluation of the prevalence and typing characteristics of APS in CeD patients within the Chinese population, filling the data gap regarding APS prevalence in CeD.

## 2 Methods

### 2.1 CeD patients and diagnosis of CeD

In this study, we retrospectively analyzed the clinical data and medical records of CeD patients diagnosed in the Department of Gastroenterology at the Xinjiang Uygur Autonomous Region People’s Hospital from March 2017 to June 2023. All the included CeD patients met the diagnostic criteria for CeD, which were based on the World Gastroenterology Organisation Global Guidelines: Celiac Disease (2017) criteria (24). Serum tissue transglutaminase (tTG) levels >20 IU/mL, and the diagnosis of CeD was confirmed after a small bowel biopsy in patients who tested positive for serum tTG and had a histopathological diagnosis suggestive of villous atrophy. All CeD patients included in this study had blood samples retained from the previous study. Individuals who did not meet the criteria for sequencing, those with incomplete clinical and case information, and those who did not consent to participate in this study were excluded from the follow-up experiments. After applying the inclusion criteria, a total of 243 CeD patients were included in this study for the screening of APS. These patients were then screened for APS-related autoimmune diseases.

The study was approved by the Ethics Committee of Xinjiang Uygur Autonomous Region People’s Hospital (Registration number: KY2023060173). All investigated patients signed an informed consent form, and the hospital ethics committee waived the requirement for informed consent for other clinical data. This study was conducted in accordance with the STROBE guidelines.

### 2.2 Diagnostic criteria for APS

The diagnostic criteria for APS in this study were based on previously published criteria (2, 11, 25, 26).

1. The diagnosis of APS-1 was established when medical records indicated the co-presence of CMC, chronic hypoparathyroidism, and AD, and a diagnosis of APS-1 was made when two of these three diseases were present. In addition, the diagnosis of APS-1 was further supported by positive screening results for serum IFNω1 and IFNα in conjunction with the mutation status of the *AIRE* gene (3).
2. The diagnosis of APS-2 was established when two of the following three disorders -AD, AITD (such as Hashimoto’s thyroiditis, Graves’ disease), and T1D at the same time. AD is a mandatory part of APS-2 and is also associated with other autoimmune gland diseases (7, 27).
3. The diagnosis of APS-3 was based on the presence of autoimmune thyroid disorders in association with other autoimmune disorders (excluding AD and hypoparathyroidism) (28).
4. There are no well-defined and widely accepted diagnostic criteria for APS-4. In the present study, the diagnosis of APS-4 was based on the approach proposed by Elisa Gatta et al, which encompassed all different clinical combinations of autoimmune diseases affecting endocrine organs that were not classified into the previous APS types (12). APS-4 was diagnosed in patients with concomitant diseases affecting one endocrine organ (excluding AD, AITD, or hypoparathyroidism) in combination with at least one other endocrine or non-endocrine organ disease.

### 2.3 IFN ω 1 and IFN α antibody detection 

Patients screened for possible APS were screened for serum IFNω1 and IFN-α antibodies.IFNω1 and IFN-α antibody test kits were purchased from Merck Reagent. Patients with positive IFNω1 and IFN-α antibodies were analyzed for *AIRE* mutations.

### 2.4 AIRE gene mutation

AIRE gene mutations were detected by whole exome sequencing. For whole exome sequencing, the concentration and quality of genomic DNA samples were first assessed using the Nanodrop 2000 and Qubit (Thermo Fisher Scientific, MA, USA). Samples were considered suitable if they had a concentration of ≥ 50 ng/μL, a total amount of ≥ 1.5 μg, and an OD260/280 ratio of 1.8 - 2.0. Subsequently, DNA was extracted from each sample for DNA library construction, and the concentration of each library was accurately determined using Qubit. All-exon capture was performed using the SureSelectXT Reagent kit (Agilent Technologies, CA, USA), followed by hybridization of the libraries to the SureSelectXT Human All Exon Kit V6 probe (Agilent Technologies, CA, USA). The libraries had an average coverage of 100-fold. Each captured library was then loaded onto the Illumina Hiseq/NovaSeq platform for high-throughput sequencing in a 2×150 bp paired-end sequencing mode to obtain whole exome sequencing results.

Based on the whole exome sequencing results, the mutation status of the *AIRE* gene was determined.

### 2.5 Thyroid autoantibody detection

All assessments of comorbidities in CeD patients in this study were based on prior medical records. In addition, we additionally tested the indicators associated with abnormal thyroid function. Abnormal thyroid function was evaluated by detecting patients’ serum total thyroxine (TT4), free thyroxine (FT4), total triiodothyronine (TT3), free triiodothyronine (FT3), thyroid - stimulating hormone (TSH), thyroglobulin antibody (TgAb), and thyroid peroxidase antibody (TPOAb). Serum TT4 and FT4 were detected using an ELISA kit, while TT3, FT3, TSH, TgAb, and TPO-Ab were detected by chemiluminescence. The normal reference ranges for TT3, TT4, FT3, FT4, and TSH were 0.8-2.0 nmol/L, 5.1-14.1 nmol/L, 2.0-4.4 pmol/L, 0.93-1.7 pmol/L, and 0.27-4.2 mU/L, respectively. TgAb levels of ≥116.0 U/mL and TPO - Ab levels of ≥35 U/mL were considered positive. Hyperthyroidism was defined as decreased serum TSH levels and elevated TT3, TT4, FT3, and FT4 levels. Hypothyroidism was defined as elevated serum TSH levels and decreased TT4 and FT4 levels. Positive TPOAb and TgAb were used to identify autoimmune thyroid disease (**Supplementary Materials**).

### 2.6 Statistical analysis

Statistical analyses in this study were performed using SPSS 24.0 software. Continuous variables following a normal distribution were presented as mean ± standard deviation (x ± s), and categorical variables were described as percentages of cases. For continuous variables that followed a normal distribution and had equal variances, independent samples t-tests were used for comparison. Categorical variables were analyzed using the chi-square (χ²) test. *P-value* < 0.05 was considered statistically significant.

## 3 Results

### 3.1 Baseline data of CeD patients

A total of 243 CeD patients were enrolled in this study, and their baseline data are presented in **Table 1**. Among the 243 enrolled CeD patients, 7 (2.9%) were children, 2 (0.8%) were adolescents, and 234 (96.3%) were adults. There were 67 (27.6%) male patients and 176 (72.4%) female patients, with a male-to-female ratio of approximately 1:2.6. Among the 243 CeD patients, 42 (17.3%) were of Han ethnicity, 97 (39.9%) were of Uyghur ethnicity, and 97 (39.9%) were of Kazakh ethnicity. There were 7 patients of other ethnicities, including 3 of Mongolian ethnicity, 1 of Hui ethnicity, 1 of Xibe ethnicity, 1 of Daur ethnicity, and 1 of Uzbek ethnicity. The BMI of the CeD patients ranged from 13.21 to 32.27, with a mean BMI of 21.5 kg/m². 63 patients (25.9%) had a BMI below 18.5 kg/m², indicating underweight, and 67 patients (27.6%) had a BMI above 24 kg/m², indicating overweight.

**Table 1:** Clinical parameters CeD enrolled in the study.

Parameters	Total (n=243)
Age	
Children(≤12 years)	7 (2.5%), 7.6 ± 2.6
Adolescents(13–17 years)	2 (0.8%), 15.5
Adults(≥18 years)	234 (96.3), 49 ± 13.5
Gender	
Male	67 (27.6%)
Female	176 (72.4%)
Nation	
Han	42 (17.3%)
Uygur	97 (39.9%)
Kazakh	97 (39.9%)
Others	7 (2.9%)
tTG -IgA (20.5-4965.5 IU/mL)	1473.8 ± 1823.2
*BMI* (13.21-32.27 kg/m^2^)	21.5 ± 4.3
Marsh Grade	
Marsh II	32 (13.2%)
Marsh IIIa	24 (9.9%)
Marsh IIIb	87 (35.8%)
Marsh IIIc	100 (41.2%)

tTG -IgA, anti tissue-transglutaminase 2 IgA;BMI, body mass index.

### 3.2 Complications of CeD patients

Based on the analysis of the previous medical records of 243 CeD patients, the prevalence of comorbidities among these patients was 78.2% (190/243) (**Table 2**). There were 21 cases (8.6%) of AITD, which consisted of 14 cases of HT and 7 cases of GD, 2 cases of AD, 80 cases (37.6%) of chronic atrophic gastritis, 4 cases (1.6%) of ulcerative colitis, 1 case of pernicious anemia, 2 cases of autoimmune hepatitis, 4 cases of T1D, 1 case of psoriasis, 1 case of dermatitis herpetiformis, 1 case of vitiligo, and 1 case of systemic lupus erythematosus. All of the above comorbidities in CeD patients could potentially be used to help diagnose APS.

**Table 2:** Complications in patients with celiac disease.

Disease	Number of patients (%)	Sex M/F	Age	Nation H/U/K	Notes
Complications of APS related diseases					
Addison disease	2 (0.8%)	0/2	36 62.4	1/1/0	APS-1/APS-2/APS-3
Hashimoto’s thyroiditis	14 (5.76%)	3/11	48.4 ± 15.8	1/6/5	APS-1/APS-2/APS-3 1Mongolian/1Uzbek
Graves’ disease	7 (2.9%)	1/6	45.3 ± 10.6	1/5/1	APS-1/APS-2/APS-3
Type 1 diabetes	4 (1.6%)	1/3	42.3 ± 18.9	1/2/1	APS-1/APS-2/APS-3/APS-4
Chronic Atrophic Gastritis	80 (37.6%)	28/52	50.6 ± 12.9	16/28/34	APS-4 1Mongolian/1Hui
Ulcerative Colitis	4 (1.6%)	0/4	50 ± 13	1/2/1	APS-4
Pernicious Anemia	1 (0.4%)	1/0	48	0/0/1	APS-4
Autoimmune Hepatitis	2 (0.8%)	0/2	57 ± 0	0/0/2	APS-4
Psoriasis	1 (0.4%)	1/0	39	0/0/1	APS-4
Herpetiform Dermatitis	1 (0.4%)	0/1	58	1/0/0	APS-4
Vitiligo	1 (0.4%)	1/0	49	1/0/0	APS-4
Systemic lupus erythematosus	1 (0.4%)	0/1	52	0/0/1	APS-4
Complications of APS unrelated diseases					
Hypothyroidism	19 (7.8%)	2/17	52.8 ± 12.1	8/3/8	
Hyperthyroidism	4 (1.6%)	0/4	55.8 ± 2.8	0/0/4	
*H. pylori* infection	44 (18.1%)	14/30	50.9 ± 13.9	8/19/13	1Hui/2Mongolian/ 1Uzbek
Osteoporosis	54 (22.2%)	14/40	47.7 ± 17.4	6/24/25	1Hui/1Daur
Osteochondrosis	2 (0.8%)	0/2	36.5 ± 6.4	0/0/2	
Anemic	48 (19.8%)	14/34	47.3 ± 15.3	4/24/18	1Xibe/1Daur
Iron Deficiency Anemia	32 (13.2%)	4/28	42.8 ± 8.8	4/11/17	
Hypoproteinemia	37 (15.2%)	14/23	49.8 ± 17	5/17/13	1Hui/1Uzbek
Hypocalcemia	35 (14.4%)	10/25	47.5 ± 11.1	2/16/15	1Uzbek/1Daur
Hypokalemia	37 (15.2%)	11/26	50.6 ± 17.3	4/13/18	1Hui/1Uzbek
Hypomagnesemia	7 (2.9%)	1/6	50.4 ± 15.7	0/4/3	
Hypophosphatemia	4 (1.7%)	1/3	46.3 ± 14.4	0/3/1	
Anxiety and Depression	25 (8.6%)	4/21	52.2 ± 14.1	5/7/12	1Mongolian
Vitamin D Deficiency	97 (39.9%)	27/70	45.9 ± 15.5	11/38/47	1Hui
Vitamin B12 Deficiency	2 (0.8%)	0/2	43.5 ± 9.2	0/2/0	
Folate Deficiency	7 (2.9%)	0/7	40.6 ± 5.2	0/4/3	
Type 2 diabetes	12 (4.9%)	4/8	65.7 ± 9.5	4/8/0	

In addition, common comorbidities of CeD included 19 cases of thyroid dysfunction (7.8%), 4 cases of hyperthyroidism (1.6%), 44 cases (18.1%) of Helicobacter pylori infection, 54 cases (22.2%) of osteoporosis, 48 cases (19.8%) of anemia, 32 cases (13.2%) of iron deficiency anemia, 37 cases (15.2%) of hypoproteinemia, 35 cases (14.4%) of hypocalcemia, 37 cases (15.2%) of hypokalemia, 21 cases (8.6%) of anxiety and depression, 97 cases (39.9%) of vitamin D deficiency, and 12 cases (4.9%) of type 2 diabetes mellitus.

### 3.3 The prevalence of different types of APS in CeD

For APS-1, all 243 enrolled individuals were screened for IFNω1 and IFN-α antibodies. The results indicated that a total of 12 patients had positive IFN-α antibody test results, and 4 patients had positive IFNω1 antibody test results. Among them, 1 individual tested positive for both IFNω1 and IFN-α antibodies. We further screened this individual for AIRE gene mutations. The results revealed that the individual harbored nine AIRE mutations: rs1133779, rs3746964, rs3214074, rs878081, rs41277548, rs1003854, rs146517804, rs941405, rs5844181, and rs3746965. This individual’s clinical comorbidities included AD, hypoparathyroidism, CeD, chronic atrophic gastritis, osteoporosis, and vitamin D deficiency. By integrating the mutation information and clinical comorbidities, we diagnosed this individual as an APS-1 patient. This was the only APS-1 case identified during this screening process. Thus, the prevalence of APS-1 in CeD was 0.4% (1/243).

APS-2 is characterized by the presence of AITD, T1D, AD, vitiligo, pernicious anemia, alopecia areata, IgA deficiency, Graves’ disease, primary hypogonadotropic hypogonadism, myasthenia gravis, and CeD, among other associated conditions. APS-2 is more prevalent than APS-1. Previous studies have indicated that APS-2 may include autoimmune thyroid dysfunction, T1D, and two out of the three criteria related to Addison’s disease, along with other autoimmune diseases. In the present study, a total of 21 patients were found to have AITD, 4 patients had T1D, and 2 patients had AD. Through the screening process, we identified only 1 patient who had all three diseases: AD, T1D, and HT. We diagnosed 1 patient as having APS-2, resulting in a prevalence of 0.4% (1/243) for APS-2 in this study cohort. In addition, this patient also had both CeD and vitamin D deficiency. Notably, previous medical records showed that this patient had been diagnosed with APS during an earlier medical visit, but the specific type of APS was not determined.

In the present study, a total of 21 patients were diagnosed with autoimmune thyroid disease, of which 14 patients had HT and 7 patients had GD. After excluding the individual who had been pre-diagnosed with APS-2, a total of 20 patients were identified as having both autoimmune thyroid disease and CeD. Therefore, in the present study, the prevalence of APS-3 among CeD patients was 8.2% (20/243), and the male-to-female ratio of these patients was 1:4.

The diagnosis of APS-4 is based on the definition from the previous study by Elisa Gatta et al (12). The clinical manifestations of APS-4 mainly include T1D or latent autoimmune diabetes in adults (LDAD), CeD, atrophic gastritis (AG), inflammatory bowel disease (IBD), pernicious anemia, autoimmune hepatitis, psoriasis, herpes-like dermatitis, vitiligo, systemic lupus erythematosus (SLE), etc. A total of 243 cases of CeD were reported. Of the 243 patients with CeD, 2 patients already diagnosed with APS-1/2 and 20 patients with APS-3 were excluded, and the remaining 221 patients were screened for APS-4. Of the 221 patients, there were 3 cases of T1D, 71 cases of atrophic gastritis, 4 cases of ulcerative colitis, 2 cases of autoimmune hepatitis, 1 case of vitiligo, 1 case of herpangina-like dermatitis, 1 case of SLE, 1 case of psoriasis, and 1 case of pernicious anemia. Based on the diagnostic definition of APS-4, 3 individuals were finally diagnosed with APS-4, namely 3 CeD patients with T1D. Their disease combinations were **(i)**: CeD, T1D, osteoporosis, and vitamin D deficiency; **(ii)**: CeD, T1D, and hypokalemia; and **(iii)**: CeD, T1D, chronic atrophic gastritis, anemia, fatty liver, and vitamin D deficiency. Thus, the prevalence of APS-4 in CeD was 1.2% (3/243).

**Figure 1:**
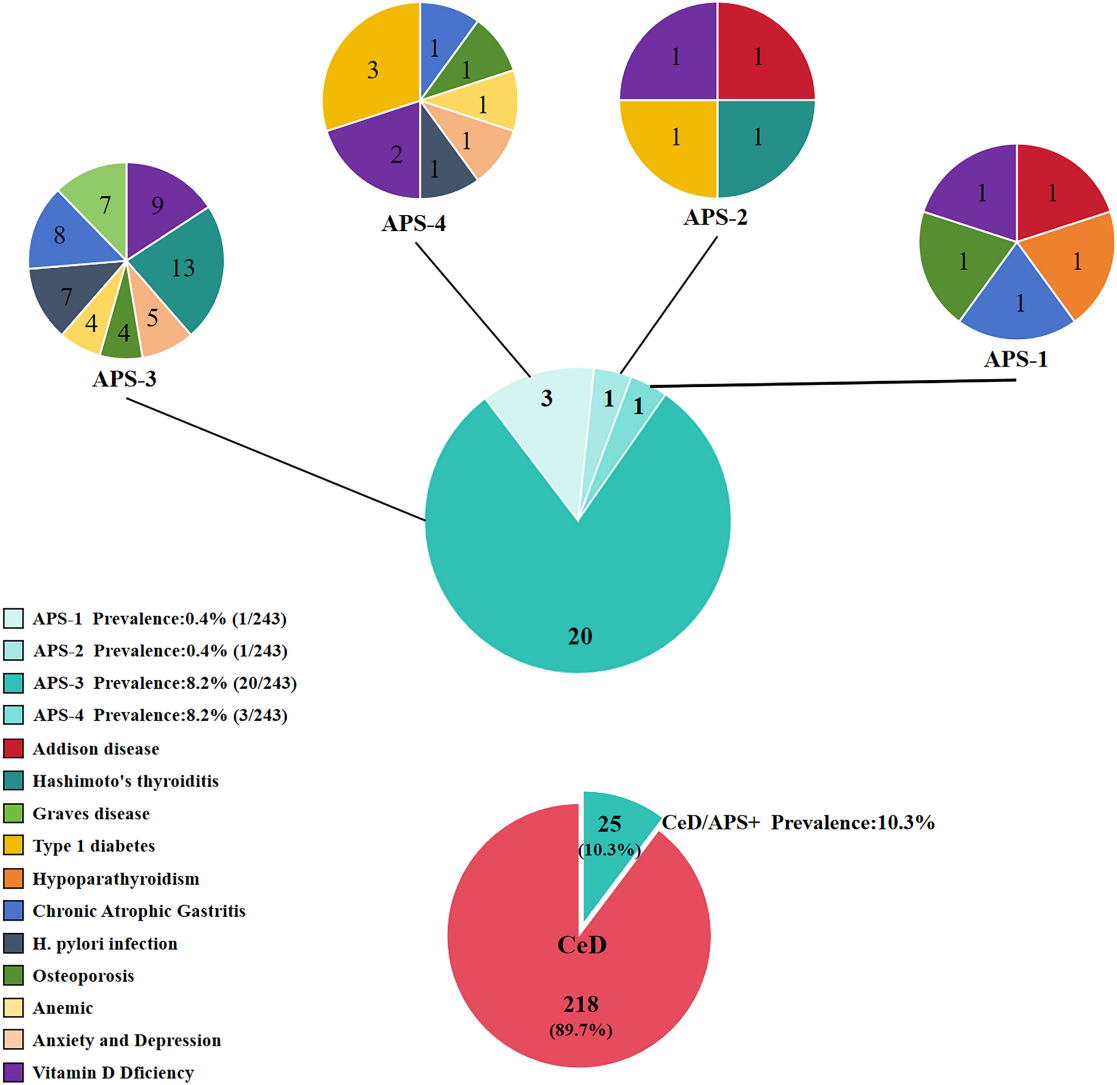
Prevalence of different types of APS and disease combinations in celiac disease.

Among the CeD patients enrolled in this study, the prevalence of APS-1 was 0.4%, that of APS-2 was 0.4%, that of APS-3 was 8.2%, and that of APS-4 was 1.2%. The overall prevalence of APS among CeD patients was 10.3% (25/243), as shown in **Figure 1**.

### 3.4 Differences in clinical characteristics between CeD combined with APS and CeD without APS

The 243 CeD patients were categorized into CeD combined APS group and CeD not combined APS group. After screening a total of 25 patients were found to be combined with APS; 218 patients were not combined with APS. Among the 25 patients with APS, common comorbidities included chronic atrophic gastritis, Helicobacter pylori infection, and vitamin D deficiency. We analyzed the differences in baseline characteristics, including age, gender, ethnicity, tTG levels, BMI, and other comorbidities, between the two groups. The results indicated that serum tTG levels were significantly lower in patients with CeD combined with APS than in patients without APS (*P* < 0.05). There were no significant differences in age, gender, ethnicity, or BMI between the two groups. Following further age stratification, middle-aged CeD patients with APS (30–60 years) were significantly older than their counterparts with CeD without APS in the same age group. In addition, we further analyzed whether there were significant differences between the two groups in terms of CeD-related comorbidities. The results showed that patients with CeD combined with APS were more likely to have hypothyroidism and experience anxiety and depression compared with those without APS (*P* < 0.05) (**Table 3**).

**Table 3:** Differences in clinical characteristics between CeD combined with APS and CeD without APS.

Parameters	CeD/APS+ (n=25)	CeD/APS - (n=218)	Statistical value	*P-*value
*Age*	47.9 ± 13.5	47.5 ± 15.5	t=0.117	0.238
Children and Adolescents (< 18 years)	1 (17 years)	8 (24.6 ± 3.3)	–	–
Youth (18–30 years)	2 (21.5 ± 3.5)	14 (24.6 ± 3.9)	t=0.505	0.585
Middle age (30–60 years)	17 (48.4 ± 7.1)	138 (45.7 ± 8.7)	t=1.160	0.035
Old age (≥60 years)	5 (63.0 ± 4.0)	43 (68.3 ± 6.3)	t=-1.802	0.151
*Gender*			χ2 = 0.800	0.371
Male	5 (20%)	62 (28.4%)		
Female	20 (80%)	156 (71.6%)		
*Nation*			χ2 = 5.382	0.146
Han	2 (8%)	40 (18.3%)		
Uygur	13 (52%)	84 (38.5%)		
Kazakh	8 (32%)	89 (40.8%)		
Others	2 (8%)	5 (2.3%)		
*tTG*	762.7 ± 1198.8	1548 ± 1878.7	t=-2.049	0.000
*BMI*	21.7 ± 5	21.5 ± 4.2	t=0.297	0.213
Complications of APS unrelated diseases				
Hypothyroidism	8 (32%)	18 (8.3%)	χ2 = 13.233	0.000
Hyperthyroidism	0	4 (1.8%)	χ2 = 0.466	0.495
*H. pylori* infection	8 (32%)	36 (16.5%)	χ2 = 3.627	0.057
Osteoporosis	6 (24%)	48 (22.0%)	χ2 = 0.051	0.821
Osteochondrosis	1 (4%)	1 (0.5%)	χ2 = 3.446	0.063
Anemic	5 (20%)	43 (19.7%)	χ2 = 0.001	0.974
Iron Deficiency Anemia	4 (16%)	28 (12.8%)	χ2 = 0.195	0.658
Hypoproteinemia	4 (16%)	33 (15.1%)	χ2 = 0.013	0.909
Hypocalcemia	5 (20%)	30 (13.8%)	χ2 = 0.704	0.400
Hypokalemia	4 (16%)	33 (15.1%)	χ2 = 0.013	0.909
Hypomagnesemia	2 (8%)	5 (2.3%)	χ2 = 2.610	0.106
Hypophosphatemia	1 (4%)	3 (1.4%)	χ2 = 0.954	0.329
Anxiety and Depression	6 (24%)	19 (8.7%)	χ2 = 5.677	0.017
Vitamin D Deficiency	13 (52%)	84 (38.5%)	χ2 = 1.696	0.193
Vitamin B12 Deficiency	1 (4%)	1 (0.5)	χ2 = 3.446	0.063
Folate Deficiency	1 (4%)	6 (2.8%)	χ2 = 0.125	0.724

## 4 Discussion

Celiac disease (CeD) is an autoimmune enteropathy precipitated by gluten intake, and its pathogenesis encompasses genetic, environmental, and immune - microenvironment factors (17, 29). Patients with CeD frequently present with comorbidities involving various glandular autoimmune disorders, such as thyroid-associated Hashimoto’s thyroiditis (HT) and Graves’ disease (GD), pancreas-associated T1D, and adrenal - associated Addison’s disease (18, 26, 30). AITD, T1D, and AD are major components of APS. The diverse comorbidities of CeD suggest a potentially increased prevalence of APS in CeD patients. Furthermore, CeD shares a common genetic background with mono-and poly-glandular autoimmune diseases, where HLA-DQ2 and/or DQ8 are the predominant shared genetic susceptibility factors. However, to date, studies investigating the incidence of APS in CeD and their clinical features remain scarce. This study systematically assessed the prevalence and typing characteristics of APS in CeD within a Chinese population for the first time, thus filling a gap in the epidemiological data.

In the present study, through the analysis of the prevalence and typing characteristics of APS among 243 CeD patients, it was found that the overall prevalence of APS in CeD patients was 10.3% (25/243), and the male-to-female ratio was 1:4. The prevalence of different types of APS was as follows: APS-1, 0.4%; APS-2, 0.4%; APS-3, 8.2%; and APS-4, 1.2%. This finding suggests that the prevalence of APS among CeD patients is significantly higher than that in the general population.

Our screening for APS-1 was based on IFNω1 and IFN-α antibody positivity in combination with *AIRE* gene mutations as the primary diagnostic approach. Subsequently, we incorporated the patients’ clinical manifestations. Specifically, the presence of at least two of the three main features - chronic cutaneous mucocutaneous candidiasis, hypoparathyroidism, and AD was used to confirm the diagnosis of APS-1 (31, 32). The 243 enrolled CeD patients were screened for IFNω1 and IFN -α antibodies. After the screening process, one individual tested positive for both IFNω1 and IFN-α antibodies. We further screened this individual for *AIRE* gene mutations and detected multiple *AIRE* gene mutations in this individual. Based on the IFNω1 and IFN-α antibody results and the *AIRE* gene test results, the diagnosis of APS-1 for this patient was confirmed. In addition to AD and hypoparathyroidism, this patient’s clinical manifestations also included CeD, chronic atrophic gastritis, osteoporosis, and vitamin D deficiency.

We identified a 19-year-old female patient with APS-2. Her tTG-IgA level was 25.8 IU/mL, and her BMI was 18.37. The patient had AD, HT, and T1D, fulfilling the diagnostic criteria for APS-2. The patient had been previously diagnosed with APS MM during an earlier endocrinology consultation, yet the specific APS type remained undetermined. In addition to these disorders, the patient had a comorbid combination of CeD and vitamin D deficiency. CeD shares a common genetic and immunological basis with APS-2. Nearly all CeD patients carry the HLA-DQ2/DQ8 haplotype, and the central role of the HLA in autoimmunity is further evidenced by the high expression of HLA-DR3/DR4 in APS-2 patients (33, 34). Regarding Addison’s disease, whether occurring in isolation or as part of APS-2, the HLA genotypes conferring the highest risk are DR3/4 and DQ2/DQ8, especially in individuals carrying the DRB1*04:04 allele (35). Furthermore, the HLA-DQA1 and HLA-DQB1 genes are strongly associated with T1D, with DQ8.1 and DQ2.5 being the principal risk haplotypes for T1D. Previous studies have also demonstrated that polymorphisms in HLA class II molecules are associated with susceptibility to APS-2, APS-3, and APS-4.In the present study, we further investigated the HLA genotype of this patient and found that the patient’s HLA typing result was DQ7.5/DQ2.2 (DQA1*05:05-DQB1*03:01/DQA1*02:01-DQB1*02:02). The DQ7.5/DQ2.2 haplotype is associated with an increased risk of developing CeD, although this risk is substantially lower than that associated with the HLA-DQ2.5 and HLA-DQ8 haplotypes (36–38). To the best of our knowledge, there have been no direct investigations into the relationship between the HLA-DQ7.5/DQ2.2 haplotype and APS. However, several studies have demonstrated that HLA genes, especially the HLA class II locus, play a crucial role in the genetic susceptibility to APS. Different HLA alleles and haplotypes may be associated with different subtypes of APS or may be protective against certain autoimmune diseases (34).

In the present study, we screened and identified 20 patients with APS - 3. Among these patients, 13 had comorbid HT and 7 had comorbid GD. AITD is one of the most prevalent comorbid glandular autoimmune disorders in CeD, and thyroid antibodies are detected in approximately 10% to 30% of CeD patients (30, 39). A study of Italian CeD patients also found that the most common comorbid autoimmune glandular disease among CeD patients was AITD, with a comorbidity rate of approximately 24.2% (8). In the present study, we found that AITD had a comorbidity rate of 8.6% among CeD patients, which is in line with previous research findings (40). The coexistence of CeD and AITD can be attributed to shared genetic factors, such as HLA-DR3 or DR4 (which are associated with DQ2 and DQ8, respectively). HLA-associated genes, particularly DQA1*0501 and DQB1*0201, are associated with both CeD and AITD (41). Considering the high comorbidity rate of AITD in CeD, the prevalence of APS-3 among CeD patients may be significantly higher than that of other APS types, and this finding was also corroborated in the present study. Notably, the implementation of a gluten-free diet (GFD) in CeD patients with AITD facilitated a reduction in the dose of thyroid medication and decelerated the progression of AITD (42).

In addition, a total of three patients with APS-4 were identified through screening, and T1D was the predominant autoimmune glandular disease among APS-4. Given the extensive heterogeneity of APS-4, patients had varying comorbidities of other autoimmune disorders in addition to gland-associated autoimmune disorders. Previous studies have found that AITD and T1D are the most prevalent autoimmune glandular comorbidities in CeD (8). A single-center study involving 111 APS-4 patients revealed that T1D served as the primary diagnostic indicator in 78% of APS-4 patients, while 19% of the subjects reported T1D as a secondary clinical manifestation of APS-4. Moreover, their study also identified CeD as the most common comorbidity other than T1D (12). This could be attributed to the fact that both diseases share common HLA susceptibility alleles (43–45). There exists a genetic and immunological overlap between T1D and CeD, especially with respect to the HLA-DQ2 and HLA-DQ8 haplotypes. HLA-related genes play a pivotal role in the immune response, which might account for the shared susceptibility of these two diseases (46). Furthermore, patients with CeD have an elevated risk of developing T1D, and the risk of developing CeD is significantly higher in T1D patients (44, 45).

Among 25 patients with APS, vitamin D deficiency (13 cases, 52%) and H. pylori infection (8 cases, 32%) were the most prevalent comorbidities apart from CeD. Vitamin D deficiency is a common comorbidity in CeD. Intestinal inflammation and injury induced by CeD can cause vitamin D malabsorption, thereby resulting in vitamin D deficiency (47, 48). Conversely, as previous studies have reported, vitamin D deficiency may exacerbate the symptoms and comorbid conditions associated with CeD. Vitamin D deficiency might be implicated in the pathogenesis of APS via multiple mechanisms. It has the potential to affect the function of immune cells, modify cytokine production, and disrupt the establishment of immune tolerance (49). Bellastella et al., in a case - control study, demonstrated that 25 - hydroxyvitamin D (25 - OHD, vitamin D) levels were significantly lower in patients with APS than in healthy subjects (*P* < 0.001) (50). In the present study, 13 out of 25 patients with APS had concomitant vitamin D deficiency, suggesting that vitamin D deficiency could be one of the clinical manifestations of APS. However, it cannot be excluded that vitamin D deficiency is a consequence of a single autoimmune disease within the APS spectrum. *H. pylori* infection was positively correlated with the development of AITD(OR = 2.25, 95%;CI: 1.72 - 2.93). *H. pylori* infection may exacerbate AITD via molecular mimicry, immune response, and other mechanisms (51, 52). It has also been demonstrated that *H. pylori* infection can impact the host’s immune system, resulting in the disruption of immune tolerance and the activation of autoimmune responses. This immune dysregulation might promote the development of multiple autoimmune diseases within the APS context (53, 54).

We further analyzed the differences between patients with CeD co-occurring with APS and those with CeD alone. The results showed that there was no significant difference in age between patients with CeD co-morbid APS and those with CeD without APS (P = 0.238). However, further stratified analysis by age groups showed that the age of patients with middle-aged CeD co-morbid APS was significantly higher than that of patients with middle-aged CeD without APS (*P* < 0.05), which may be mainly attributed to the fact that the prevalence of APS-associated autoimmune diseases usually increases with age. A large-scale UK cohort study (22 million participants) on age- and sex-standardized incidence and prevalence of 19 autoimmune diseases showed that approximately 4.4% of individuals were newly diagnosed with at least one autoimmune disease, with a mean diagnosis age of 54 years, suggesting that middle age (30–60 years) may represent a high-prevalence age range for autoimmune disease development (55). In addition, the levels of tTG in patients with CeD co - occurring with APS were significantly lower than those in patients with CeD without APS comorbidity (*P* < 0.001), suggesting that the tTG level in CeD patients may not be associated with the incidence of autoimmune diseases. A prior study also indicated that not all patients with the highest anti - tTG antibody titers had concomitant autoimmune diseases (20). Besides age and tTG, no statistically significant differences were observed between the CeD with APS comorbidity group and the CeD without APS comorbidity group in terms of gender, ethnicity, and BMI (*P* > 0.05). Moreover, we discovered that patients with CeD co-occurring with APS were more prone to experience anxiety and depression compared to patients with CeD alone (*P* > 0.05). This might be related to several factors, such as the autoimmune processes of diverse autoimmune diseases, hormonal imbalances, and the reduced quality of life associated with chronic diseases (56, 57).

Although this study addresses a gap in data regarding the prevalence of APS among Chinese patients with CeD, it has several limitations. Firstly, the current study was a single-center retrospective investigation. The retrospective nature of the study design might have introduced sample selection bias. Secondly, the identification of comorbidities in CeD patients in the present study was derived from hospital-based medical records in the past. Since the patients were not followed up, the study cannot determine the sequence of occurrence of different autoimmune diseases. Moreover, the ethnic distribution of CeD patients included in the present study was concentrated in the Uyghur and Kazakh ethnic groups (79.8%). It is plausible that certain geographic or genetic factors might influence the prevalence of APS. Nevertheless, our meticulous diagnostic procedures for various types of APS and the analysis of patients’ clinical records enhanced the reliability of our results.

In conclusion, the overall prevalence of APS among CeD patients was 10.3%. The prevalence of different subtypes of APS was as follows: APS-1, 0.4%; APS-2, 0.4%; APS-3, 8.2%; and APS-4, 1.2%. To the best of our knowledge, this study is the first to report the prevalence of APS in CeD patients. The high prevalence of AITD among CeD patients might be the primary factor contributing to the co-occurrence of APS in these patients. Moreover, CeD and APS share a genetic background, and both are associated with major histocompatibility complex (MHC) class II molecules. This suggests the significance of screening for APS in CeD patients. Our study also revealed that CeD patients with concomitant APS were more likely to experience anxiety and depression. These findings not only disclose the unique association pattern between CeD and APS in the Chinese population but also offer a crucial foundation for clinical screening. In the future, large-scale cohort studies are required to further explore the association and shared pathogenesis between CeD and gland-related autoimmune diseases. Moreover, screening for APS in CeD patients is essential for the early detection and treatment of associated autoimmune diseases and the enhancement of patients’ quality of life.

## Data Availability

The original contributions presented in the study are included in the article/**Supplementary Material**. Further inquiries can be directed to the corresponding author.
